# New Inventions

**Published:** 1895-07

**Authors:** 


### NEW INVENTIONS.

An automatic watering-trough.

An animal-poke with an articulated poke-bar to fasten to the animal, with crown, brow and nose bands, also a neck and back strap, allowing a certain restricted movement of the head.

A veterinary canula, with a series of spring-fingers adapted to spread apart by their own elasticity, made to hold liquids and expel them as desired.

A new animal-shears with comb attachment.

Horse-training harness with one or more body-straps and two or more leg-straps, with double snap-hook fastenings.

Horseshoer's stand for securing the foot when shoeing.

A four-sided bottle, two sides of which are rough as warning projections, the skull and cross-bones below on one side, and poison in raised letters on another.

A bridle-bit, with main bit to which check-bit is attached by a series of independent lace-cord connections, forming a yielding tongue-barrier.

A thermocautery with improved perforated diaphragm.

A driving-bit, with extension-strap between nostril to a curved part around the upper lip, and attached at each side to the rings of the bit.

An adjustable knock-down horse-stall.

### POSITION TO EXCHANGE.

A TWELVE-HUNDRED-DOLLAR a year army position, carrying with it allowances of quarters, light, and fuel, with privilege of outside practice, for an equally remunerative position or practice in or near a city.

A pleasant station, delightful climate, especially beneficial to invalids; splendid hunting and fishing. Address "Exchange," care of The Journal of Comparative Medicine and Veterinary Archives, 3452 Ludlow Street, Philadelphia, Pa.

